# Split-CreERT2: Temporal Control of DNA Recombination Mediated by Split-Cre Protein Fragment Complementation

**DOI:** 10.1371/journal.pone.0008354

**Published:** 2009-12-16

**Authors:** Johannes Hirrlinger, Robert P. Requardt, Ulrike Winkler, Franziska Wilhelm, Christine Schulze, Petra G. Hirrlinger

**Affiliations:** 1 N05 Neural Plasticity, Interdisciplinary Centre for Clinical Research (IZKF), University of Leipzig, Leipzig, Germany; 2 Carl-Ludwig-Institute for Physiology, University of Leipzig, Leipzig, Germany; 3 Paul-Flechsig-Institute for Brain Research, University of Leipzig, Leipzig, Germany; Institute of Infectious Disease and Molecular Medicine, South Africa

## Abstract

**Background:**

DNA recombination technologies such as the Cre/LoxP system advance modern biological research by allowing conditional gene regulation *in vivo*. However, the precise targeting of a particular cell type at a given time point has remained challenging since spatial specificity has so far depended exclusively on the promoter driving Cre recombinase expression. We have recently established split-Cre that allows DNA recombination to be controlled by coincidental activity of two promoters, thereby increasing spatial specificity of Cre-mediated DNA recombination. To allow temporal control of split-Cre-mediated DNA recombination we have now extended split-Cre by fusing split-Cre proteins with the tamoxifen inducible ERT2 domain derived from CreERT2.

**Methodology/Principal Findings:**

In the split-CreERT2 system, Cre-mediated DNA recombination is controlled by two expression cassettes as well as the time of tamoxifen application. By using two independent Cre-dependent reporters in cultured cells, the combination of NCre-ERT2+ERT2-CCre was identified as having the most favorable properties of all constructs tested, showing an induction ratio of about 10 and EC_50_-values for 4-hydroxy-tamoxifen of 10 nM to 70 nM.

**Conclusions/Significance:**

These characteristics of split-CreERT2 *in vitro* indicate that split-CreERT2 will be well suited for inducing DNA recombination in living mice harboring LoxP-flanked alleles. In this way, split-CreERT2 will provide a new tool of modern genetics allowing spatial and temporal precise genetic access to cell populations defined by the simultaneous activity of two promoters.

## Introduction

DNA recombination technologies such as the Cre/LoxP system have advanced and refined the analysis of gene and cell functions in mice [1]–[4]. In contrast to classical *in vivo* “knockout” strategies, which result in a complete deletion of gene function in the whole organism, this conditional gene targeting technology enables a cell type-specific deletion of genes by driving the expression of Cre recombinase under the control of a cell type-specific promoter [1], [5], [6]. In addition, the Cre/LoxP system has been used for fate mapping and for cell ablation *in vivo* [7]–[12].

Despite the enormous success of cell-specific gene targeting using Cre/LoxP, two major limitations are inherent to this system: 1) the time of recombination is solely determined by the time of onset of the promoter activity driving Cre expression and can not be controlled experimentally; 2) the specificity of the promoter used to drive Cre expression is often not sufficient to selectively target a specific cell population. To overcome the first limitation, inducible forms of the Cre recombinase were developed to allow for temporally controlled somatic mutagenesis thereby circumventing problems arising from constitutive Cre recombinase activity [2]. The most widely used version of inducible Cre is CreERT2 [13], in which Cre is fused to a mutated ligand binding domain of the human estradiol receptor (ERT2), which does not bind endogenous estradiol but is highly sensitive to nanomolar concentrations of tamoxifen or its metabolite 4-hydroxy-tamoxifen (4OHT) [13]. In stably transfected F9 murine embryonal carcinoma cells, the EC_50_ for 4OHT was found to be 6 nM [13]. In the absence of the inducer tamoxifen, the fusion protein is trapped in the cytosol by binding to heat shock proteins. Administering the synthetic ligand tamoxifen releases CreERT2 from this complex, allowing CreERT2 to enter the nucleus and excise LoxP-flanked DNA regions [2].

An enhanced precision of spatial control of DNA recombination can be achieved by controlling Cre expression and activity not by a single promoter, but by the combination of two promoters enabling the more precise genetic targeting of a distinct cell type [14]. Following this rational, we recently developed the “split-Cre system” based on the functional complementation of Cre protein fragments [15]. The coding sequence of Cre recombinase was divided into two parts (NCre and CCre) which were both fused to constitutive GCN4 protein-protein interaction domains. Placing both split-Cre proteins under the control of two different promoters, we observed DNA recombination in the brain of living transgenic mice in a cell population, which was defined by the simultaneous activity of the human glial fibrillary acidic protein promoter as well as the mouse proteolipid protein promoter [15].

In the present work, we combined the temporal control by an inducible Cre with the precise spatial control of split-Cre. We have developed a conditional and inducible DNA recombination system for cell populations defined by the simultaneous activity of two promoters. The ERT2 tamoxifen binding domain of CreERT2 was added to the split-Cre system to achieve tamoxifen inducibility of split-Cre mediated DNA recombination. This strategy was named split-CreERT2. We show that after transfection of cultured cells, split-CreERT2 allows induction of DNA recombination by 4OHT with little activity in the absence of 4OHT. Thus, split-CreERT2 combines temporal control and two-dimensional spatial control of DNA recombination and should enable further refinement of the analysis of gene and cell function in living mice and other organisms.

## Results

**Figure 1:**
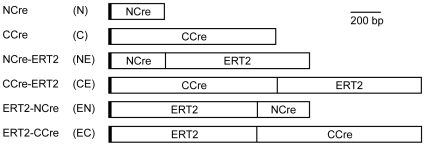
Split-CreERT2 constructs. The original, non-inducible split-Cre proteins NCre and CCre have recently been described [15]. To generate NCre-ERT2 and CCre-ERT2, the ERT2-domain was fused to the C-terminus of NCre and CCre, respectively. Vice versa, the ERT2-domain was fused to the N-terminus of NCre and CCre to obtain ERT2-NCre and ERT2-CCre, respectively. The abbreviations for the different constructs used in this paper are given in brackets. The thick vertical line at the N-terminus of all constructs denotes the position of the corresponding immunotag (NCre containing constructs: Flag-tag; CCre containing constructs: Myc-tag).

**Figure 2:**
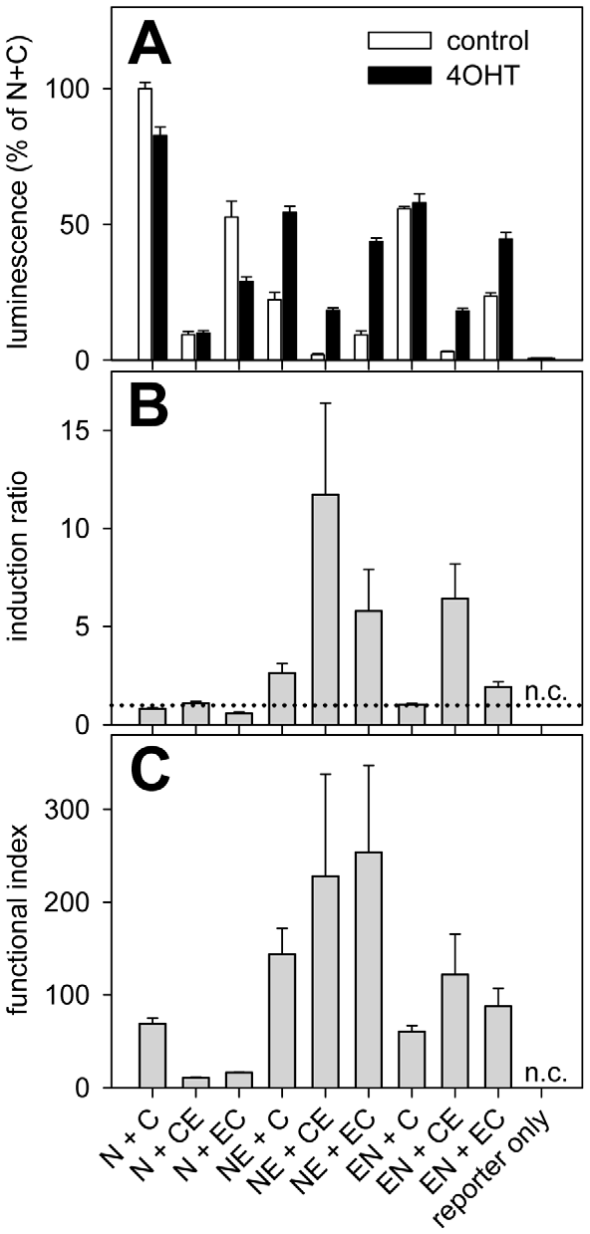
Split-CreERT2 mediated DNA recombination analyzed in CHO cells by luciferase assays. A: CHO cells cultured in 96-well cell culture plates were transfected with the different combinations of split-CreERT2 plasmids (for abbreviations see Fig. 1; 100 ng of each plasmid/well) and cultured in the absence (control, open bars) or presence of 4OHT (1 µM, black bars). Cre-dependent DNA recombination was analyzed by cotransfection of a CMV-LoxP-STOP-LoxP-luciferase reporter plasmid. Luciferase activity was normalized to luciferase activity in N+C transfected cells cultured in the absence of 4OHT. B: Induction ratios were calculated as [(luciferase activity in the presence of 4OHT)/(luciferase activity in the absence of 4OHT)]. The dotted line denotes a ratio of 1, which means no induction. C: A functional index of each combination of split-CreERT2 proteins was calculated as [(induction ratio)×(luciferase activity in the presence of 4OHT)]. This functional index is highest if the combination of split-CreERT2 proteins shows high inducibility and high activity in the presence of 4OHT. The figure shows data from 3 independent experiments, each of which was performed at least in triplicate. n.c.: not calculated.

**Figure 3:**
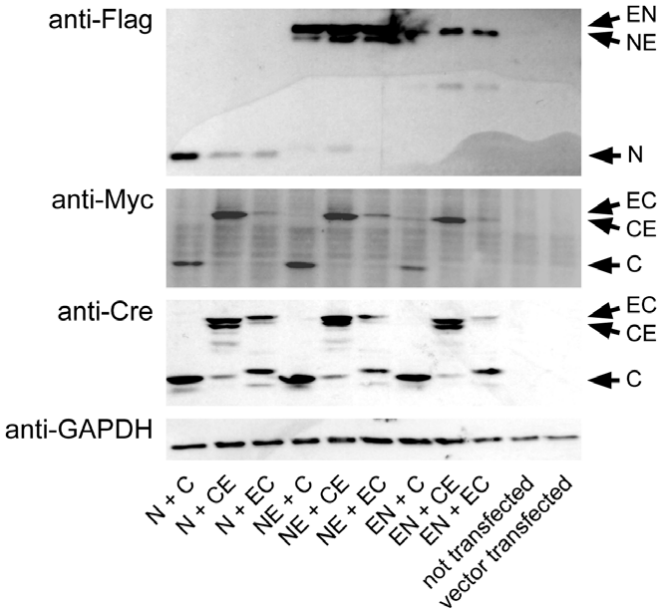
Western Blot analysis of the expression of the different split-CreERT2 constructs. CHO cells were transfected with the different combinations of split-CreERT2 plasmids as indicated. Expression of N, NE and EN was analyzed by immunoblotting with anti-Flag antibodies, whereas C, CE and EC were detected by anti-Myc as well as anti-Cre antibodies. GAPDH was used as a loading control. The observed sizes of the proteins were as expected (N: 14.8 kDa; NE: 50.1 kDa; EN: 51.1 kDa; C: 41.6 kDa; CE: 77.0 kDa; EC: 78.0 kDa; GAPDH: 35.9 kDa).

Based on our previous results with functional complementation of split-Cre proteins [15], we constructed inducible versions of split-Cre proteins by fusing the tamoxifen inducible ERT2-domain to each of the termini of both NCre and CCre (Fig. 1). The “split-CreERT2” proteins obtained were termed NCre-ERT2, CCre-ERT2, ERT2-NCre or ERT2-CCre (Fig. 1). We refer to these constructs below using the abbreviations NE, CE, EN and EC, respectively (the original NCre and CCre [15] will be referred to as N and C, respectively; Fig. 1). In a first attempt to determine whether complementation of these fusion proteins can reconstitute Cre-dependent DNA recombination activity and whether this activity is dependent on tamoxifen application, CHO cells were transfected with combinations of split-Cre and split-CreERT2 proteins and incubated in the absence or presence of 4OHT. To detect DNA recombination a CMV-LoxP-STOP-LoxP-firefly luciferase reporter plasmid was cotransfected and luciferase activity was measured (Fig. 2A). Luciferase activity was normalized to luciferase activity in N+C transfected cells cultured in the absence of 4OHT, which was set as 100%. Cells transfected with full-length Cre recombinase showed 234±25% (n = 10) of the activity of N+C, confirming our previous results [15]. In contrast, all cells transfected with ERT2-tagged proteins showed a lower luciferase activity even after application of 4OHT compared to N+C transfected cells and the different combinations of split-CreERT2 proteins showed unequal activity (Fig. 2A). Western Blot analysis revealed expression of all constructs (Fig. 3), suggesting that the differences in activity are due to functional differences and not due to lack of expression. Nevertheless, several combinations of split-CreERT2 proteins showed inducibility of DNA recombination by 4OHT (Fig. 2A) and an induction ratio was calculated as [luminescence in the presence of 4OHT/luminescence in the absence of 4OHT] (Fig. 2B). This analysis revealed that the combination of NE+CE, NE+EC and EN+CE can be induced best by 4OHT (Fig. 2B). However, while e.g. NE+CE shows the highest inducibility (Fig. 2B), the total activity even in the presence of 4OHT is low (Fig. 2A). Therefore, a functional index was defined as [induction ratio×luminescence in the presence of 4OHT] to asses which combination of split-CreERT2 proteins combines the favorable properties of high activity and high inducibility (Fig. 2C). This analysis indicated that NE+CE and NE+EC are the best combinations (Fig. 2C).

**Figure 4:**
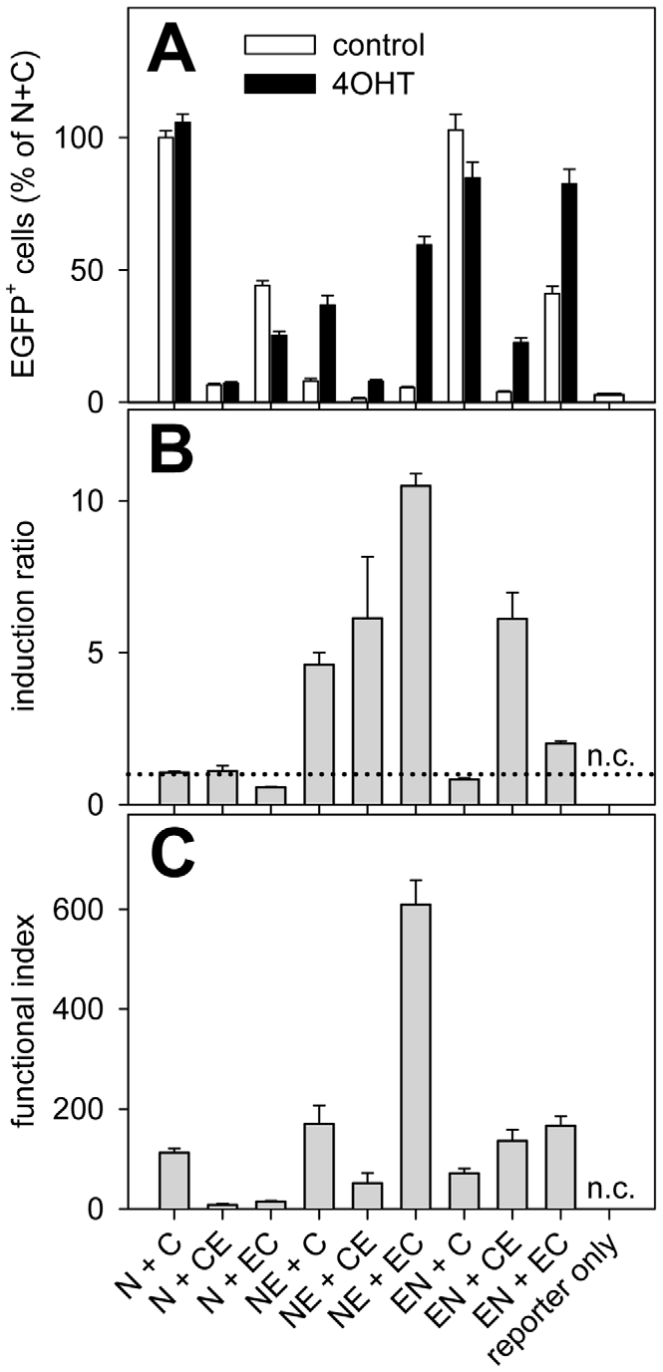
Split-CreERT2 mediated DNA recombination analyzed in PC12 20.4 cells by flow cytometry. A: PC12 20.4 cells, which harbor a stably transfected CMV-LoxP-STOP-LoxP-EGFP reporter cassette, were cultured in 24-well cell culture plates and transfected with the different combinations of split-CreERT2 plasmids (for abbreviations see Fig. 1; 400 ng of each plasmid/well) and cultured in the absence (control, open bars) or presence of 4OHT (1 µM, black bars). B: Induction ratios were calculated as in Fig. 2. The dotted line denotes a ratio of 1, which means no induction. C: A functional index of each combination of split-CreERT2 proteins was calculated as indicated in the legend of Fig. 2. The figure shows data from 8 independent experiments, each of which was performed at least in triplicate. n.c.: not calculated.

Transgenic mice used for conditional gene targeting experiments normally harbor only a few LoxP-recombination sites, which are stably integrated within the genomic DNA. However, in our luciferase reporter experiments the reporter plasmids were transiently transfected resulting in a high number of LoxP-sites localized on plasmids. To study split-CreERT2-mediated recombination in a cell culture system containing only a few LoxP-sites stably integrated within the genomic DNA, resembling more realistically the situation in transgenic mice, PC12 20.4 cells were used [16]. PC12 20.4 cells contain a stably integrated CMV-LoxP-STOP-LoxP-EGFP cassette and report Cre-dependent DNA recombination by expression of EGFP [16]. These cells were transfected with the different combinations of split-CreERT2 plasmids, incubated in the absence and presence of 4OHT and split-CreERT2 dependent DNA recombination was analyzed using flow cytometry (Fig. 4A). Cells transfected with full-length Cre recombinase showed 219±13% (n = 27) of the activity of N+C, again confirming our previous results [15]. The different combinations of split-CreERT2 proteins showed variable levels of activity (Fig. 4A) and inducibility (Fig. 4B) similar to the results obtained in CHO cells transiently transfected with the reporter plasmids (Fig. 2). The calculation of the functional index (Fig. 4C) indicated that NE+EC clearly combines the most favorable properties, thereby confirming the data obtained by luciferase assays in CHO cells. Interestingly, NE+CE showed less activity in PC12 20.4 cells than in CHO cells, resulting in a low functional index despite substantial inducibility.

**Figure 5:**
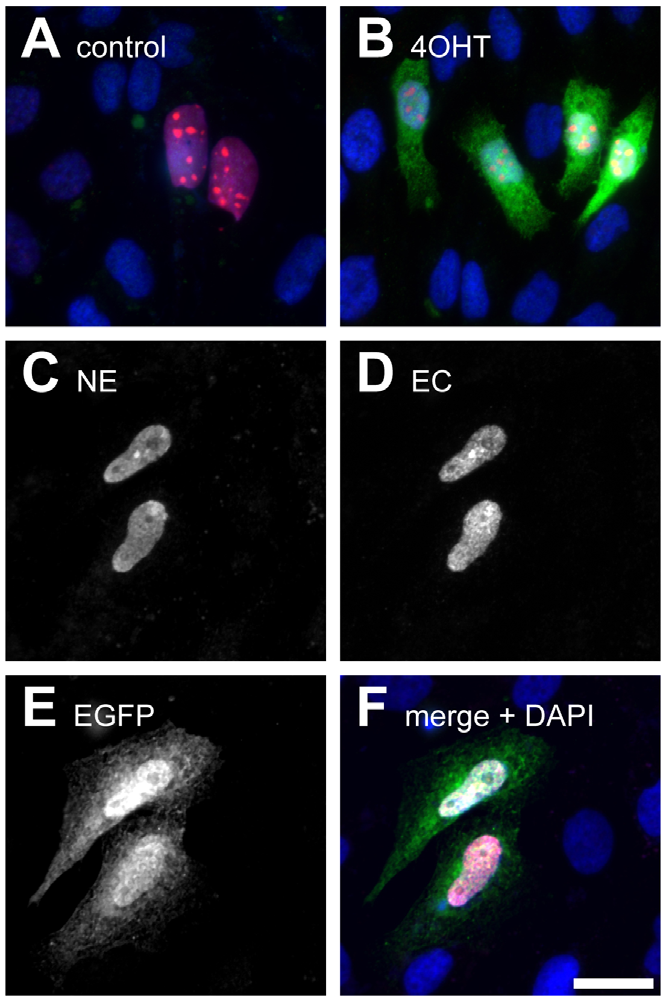
Immunocytochemical analysis of the split-CreERT2 protein combination NCre-ERT2 + ERT2-CCre (NE+EC). A, B: CHO cells cultured in 24-well cell culture plates were transfected with NE+EC (150 ng of each plasmid/well) along with a reporter plasmid which expresses EGFP only after Cre-mediated DNA recombination (400 ng/well) and an expression vector coding for nuclear DsRed (100 ng/well; shown in red). Cells were cultured in the absence (A) or presence of 4OHT (1 µM, B). Recombination is detected by EGFP reporter expression (green; visualized by immunostaining using anti-GFP-antibodies). In blue, DAPI-staining of all nuclei is shown. C–F: Immunocytochemical confirmation of expression of NE and EC and of recombination in NE+EC transfected cells after application of 4OHT. CHO cells were transfected with NE+EC (400 ng of each plasmid/well) along with a reporter plasmid which expresses EGFP only after Cre-mediated DNA recombination (400 ng/well) and cultured in the presence of 4OHT (1 µM). NE (C), EC (D) and EGFP (E) were visualized by immunostaining for Flag-tag, Myc-tag or GFP, respectively, and detected by Cy5-, Cy3- or Cy2-conjugated secondary antibodies. F shows the merged images with NE in magenta, EC in red, EGFP in green and DAPI in blue. Note the additional nuclei stained with DAPI but not by anti-Flag- or anti-Myc-antibodies, which are also negative for EGFP. The bar in F corresponds to 20 µm and applies to all panels.

Based on these results, we further characterized the properties of the combination NE+EC. CHO cells were transfected with NE+EC along with a CMV-LoxP-STOP-LoxP-EGFP-reporter plasmid and a vector expressing nuclear DsRed as a transfection control (Fig. 5A, B). In the absence of 4OHT, transfected cells could readily be identified by nuclear staining with DsRed, however, no EGFP could be detected (Fig. 5A). In contrast, after application of 4OHT (1 µM), DsRed-positive cells were also positive for EGFP indicating successful recombination of the reporter plasmid (Fig. 5B). Furthermore, expression of NE and EC was analyzed by immunocytochemical staining for the Flag- and Myc-tag, respectively (Fig. 5C–F). After application of 4OHT, cells expressing both NE and EC could be identified by nuclear localization of the respective signals for the Flag- and Myc-tag (Fig. 5C, D). Most of these cells also expressed EGFP (Fig. 5E). Quantitative analysis revealed that 86.1% of cells positive for both NE and EC were also positive for EGFP (n = 122 cells analyzed from 2 independent stainings).

**Figure 6:**
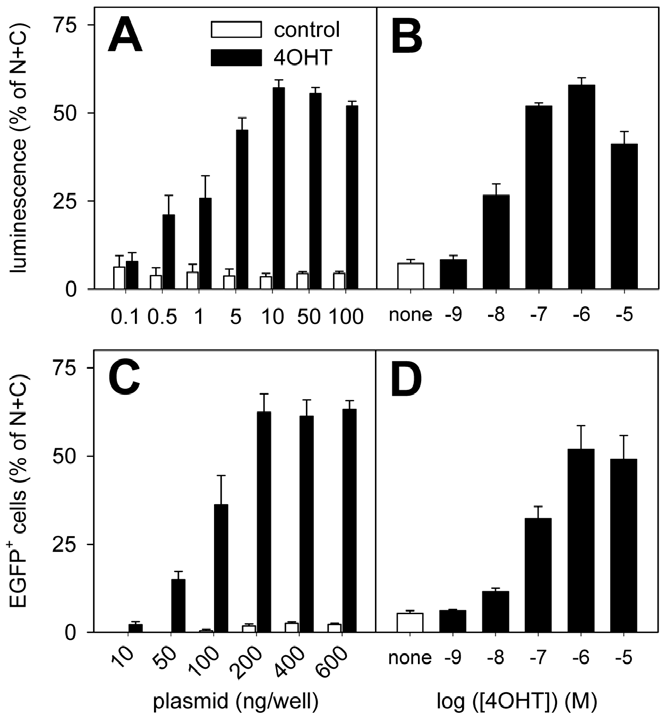
Detailed characterization of the split-CreERT2 protein combination NCre-ERT2 + ERT2-CCre (NE+EC). A: CHO cells were transfected with different amounts of NE+EC and cultured in the absence (control, open bars) or presence of 4OHT (1 µM, black bars). Recombination was analyzed by luciferase reporter expression. The luminescence of reporter-only transfected cells (1.8±0.3%; n = 15) was subtracted from all values. B: CHO cells were transfected with 100 ng each of NE+EC and cultured in the presence of different concentrations of 4OHT. Luminescence in A and B was normalized to the luminescence observed in N+C transfected cells (100 ng of each plasmid/well) in the absence of 4OHT, which was set as 100%. C: PC12 20.4 cells were transfected with different amounts of NE+EC and cultured in the absence (control, open bars) or presence of 4OHT (1 µM, black bars). Recombination was analyzed by EGFP reporter expression using flow cytometry. The percentage of EGFP^+^-cells observed in the absence of NE+EC (4.4±0.7%; n = 9) was subtracted from all values. D: Cells were transfected with NE+EC plasmids (400 ng each/well) and cultured in the presence of different concentrations of 4OHT. The number of EGFP-positive cells in C and D was normalized to the number of EGFP-positive cells in N+C transfected cultures (400 ng of each plasmid/well) in the absence of 4OHT, which was set as 100%. All panels summarize data from a minimum of 3 independent experiments, each of which was performed at least in triplicate.

To analyze the dose-response relationship for both the amount of transfected plasmid DNA and for the concentration of 4OHT, we performed luciferase assays on CHO cells (Fig. 6A, B) and flow cytometry on PC12 20.4 cells (Fig. 6C, D). First, cells were transfected using different amounts of NE and EC plasmids and incubated in the presence or absence of 4OHT (Fig. 6A, C). In the absence of 4OHT, only minor reporter gene expression could be detected with the plasmid concentrations used. In contrast, in the presence of 4OHT, a dose dependent increase in reporter gene expression was observed (Fig. 6A, C). From this data, the EC_50_ value for the amount of transfected plasmids was calculated as 1.4±0.7 ng/well and 89±56 ng/well (n = 3–5 experiments each of which was performed in triplicate) for luciferase and flow cytometry assays, respectively. Similarly, a dose dependent increase in reporter gene expression was observed with increasing concentrations of 4OHT (Fig. 6B, D). From these data, the EC_50_ value for 4OHT was calculated as 10±5 nM and 68±50 nM for luciferase and flow cytometry assays, respectively (n = 3 experiments each of which was performed in triplicate. The values obtained from luciferase and flow cytometry assays are not significantly different, p = 0.313, t-test).

## Discussion

In the current work, split-CreERT2 is presented as a new tool adding temporal control of DNA recombination to the recently described split-Cre system [15]. The ERT2-domain [13] was fused to the N-terminus or the C-terminus of the constitutively active NCre- and CCre-proteins [15]. Three parameters were assessed to dissect the functional properties of split-CreERT2 proteins: 1) low recombination in the absence of tamoxifen (no leakage); 2) high activity of recombination in the presence of tamoxifen; 3) a high induction ratio. When the different combinations of split-CreERT2 proteins were analyzed, not all versions showed the same properties regarding these criteria. Generally, the reporter gene expression from split-CreERT2 proteins was lower than that from the non-inducible forms of split-Cre (N+C; Fig. 2A, 4A), indicating lower recombination activity of split-CreERT2 compared to split-Cre in cell culture, which might hamper the efficient targeting of cells in living mice. However, a similarly reduced recombination activity of split-Cre was observed when compared to full-Cre *in vitro* [15]. Nevertheless, in transgenic mice split-Cre induced robust reporter gene activation, which was probably more limited by mosaic transgene expression rather than by split-Cre efficiency itself [15]. These findings may indicate that the situation *in vitro* and *in vivo* differs e.g. in expression kinetics, expression levels, kinetics of activation and DNA recombination. Whether the lower DNA recombination activity of split-CreERT2 in cell culture will limit DNA recombination in mice *in vivo* remains to be determined experimentally by generating appropriate transgenic mice.

The maximal induction ratio observed for split-CreERT2 was about 10 (Fig. 2B, 4B), which is in the same range as the reported *in vitro* induction ratios of CreERT and CreERT2 (about 7 to 15; [13], [17]). As both CreERT and CreERT2 have been used successfully in mice *in vivo*, this suggests that the induction ratios found for split-CreERT2 will be sufficient for successful use in living animals. However, while a high induction ratio is necessary for successful use of split-CreERT2, a high total activity in the presence of 4OHT is also needed. For this reason, we calculated a “functional index” as the product of induction ratio and activity (Fig. 2C, 4C). This analysis indicated that the split-CreERT2 variants NE+EC performed best.

The lack of recombination activity from the combination of N+CE was unexpected since the fusion of ERT2 to the C-terminal end of CCre is highly comparable to the well established CreERT2 protein. The cloning strategy resulted only in the exchange of three amino acids for two (LEP→TS) in the region connecting the Cre coding sequence to the ERT2 coding sequence. However, the other combinations of CE (with NE and EN) also showed very little recombination activity. This suggests that the ERT2 domain on the C-terminus of CCre might spatially interfere with the GCN4 protein-protein interaction domain of the NCre constructs or of CCre-ERT2 itself, as this interaction domain is not present in CreERT2 [13]. In contrast, in the case of N+EC, application of 4OHT actually decreased reporter gene activity, which was already high in the absence of 4OHT. The high basal reporter gene activity might indicate that EC is not completely trapped within the cytosol in the absence of 4OHT, thereby allowing functional complementation with N in the nucleus even in the absence of 4OHT. However, the reason for the decrease of reporter gene activity after application of 4OHT remains obscure. Most likely, the conformational change in EC induced by binding of 4OHT impairs the interaction with N, thereby reducing functional complementation and DNA recombination activity.

The successful use of split-CreERT2 in living mice will require that tamoxifen reaches the targeted cells in sufficient concentrations to induce split-CreERT2 nuclear translocation by binding to the ERT2-domain. An EC_50_-value of about 10 nM to 70 nM 4OHT for luciferase and flow cytometry assays, respectively, was determined for NE+EC. While the concentration of tamoxifen or 4OHT within tissues after application of tamoxifen has not been determined in mice *in vivo*, indirect evidence indicates that tamoxifen concentrations will not be limiting. For the well established CreERT and CreERT2, an EC_50_ of 20 nM and 6 nM 4OHT was determined in *in vitro* assays similar to those used here [13]. In addition, by analyzing DNA recombination directly by Southern blotting, EC_50_ values>100 nM have been determined in embryonic stem cells for CreERT and CreERT2 [17]. As these EC_50_ values of CreERT and CreERT2 are within the same range as determined for split-CreERT2 and both of these inducible Cre versions have been used successfully in mice *in vivo* (for example: CreERT: [18]–[22]; CreERT2: [22]–[28]; for overview: [29]), the tissue concentrations of tamoxifen will most likely be sufficient for efficient induction of split-CreERT2.

Split-CreERT2 allows controlling DNA recombination spatially by the simultaneous activity of two promoters and temporally by application of tamoxifen. Similar criteria are met by another inducible recombination system based on Cre protein fragment complementation [30], [31]. In this approach, the dimerization of Cre fragments is regulated by rapamycin dependent interaction of FKBP- and FRB-protein domains. When expressed in mice driven by two constitutively active promoters, partial DNA recombination could be observed in several tissues including liver, kidney and heart. However, no recombination was observed in brain, muscle or epidermis [31]. At least for the brain, this might well be due to the poor blood-brain barrier permeability of rapamycin. In addition, rapamycin might have several side effects due to its function as immunosuppressant. Split-CreERT2 proteins are supposed to interact constitutively when co-expressed within a cell due to the constitutively active GCN4 protein-protein interaction domain [15], but the translocation to the nucleus is regulated by tamoxifen as described for CreERT2 [23], [24]. While tamoxifen might also have some side effects via various mechanisms [32]–[35] it has already been used successfully to induce DNA recombination in numerous tissues including the brain [29]. This profound experience with experimental strategies for tamoxifen application, with kinetics of recombination, tissue specificity etc. will facilitate the transfer of the split-CreERT2 system into mice *in vivo*. While the function of split-CreERT2 in living mice remains to be demonstrated, it is very likely that split-CreERT2 - given this enormous potential - will be a significant addition to the toolbox of modern mouse genetics.

## Materials and Methods

### Cloning

The pCMV-Tag2B-NCre- and pCMV-Tag3B-CCre-plasmids have been recently described in detail [15]. To generate pERT2-NCre and pERT2-CCre, a PCR was performed using pCreERT2 as template and the primers 5′-GAGAGGATCCTCTGCTGGAGACATGAGAGC-3′ and 5′-GAGAGGATCCGCTGCCGTTGCTGGCGCCAGGGTTGCTAGGGCTGGCGACTGTGGCAGGGAAACCC-3′. BamHI restriction sites were added to the 5′- and 3′-end of the coding sequence of the ERT2-domain by PCR. In addition, a linker sequence (amino acid sequence ASPSNPGASNGS) [15] was added to connect the ERT2-domain and the NCre- or CCre part of the fusion protein. The PCR product was cloned into the BamHI restriction site of pCMV-Tag2B-NCre and pCMV-Tag3B-CCre (all restriction enzymes used were from Fermentas, St.Leon-Rot, Germany).

To generate pNCre-ERT2 and pCCre-ERT2-constructs, the Stop codon within pCMV-Tag2B-NCre- and pCMV-Tag3B-CCre-plasmids was removed and a SpeI restriction site was introduced at the 3′-end of the NCre- or CCre open reading frame. To do this, PCR was performed using the primers 5′-GAGAGAATTCAGCGGCCCTCCCA-3′ and 5′-GAGAAAGCTTACTAGTGTTCAGCTTGCACCAGGCA-3′ and pCMV-Tag2B-NCre as template. The PCR product was digested using EcoRI and HindIII and cloned into the EcoRI/HindIII restriction sites of pCMV-Tag2B-NCre. To modify pCMV-Tag3B-CCre accordingly, primers 5′-TCCACAGCTGGTGTGGAGAA-3′ and 5′-GAGAAAGCTTACTAGTGTCCCCATCCTCGAGCAG-3′ were used with pCMV-Tag3B-CCre as template. The PCR-product was digested using BstEII and HindIII and cloned into the BstEII/HindIII restriction sites of pCMV-Tag2B-NCre. Finally, a PCR was performed using primers 5′-GAGAACTAGTTCTGCTGGAGACATGAGAGC-3′ and 5′-GAGAACTAGTTCAGACTGTGGCAGGGAAACCC-3′ with pCreERT2 as template to add a SpeI restriction site to both ends of the ERT2 domain as well as a Stop codon to the 3′-end of the ERT2 open reading frame. This PCR product was digested using SpeI and cloned into the SpeI restriction site introduced as described above at the 3′-end of pCMV-Tag2B-NCre- and pCMV-Tag3B-CCre-open reading frames. All constructs were verified by restriction analysis and sequencing.

### Cell Culture, Transfection and Analysis of Recombination

CHO cells (DSMZ, Braunschweig, Germany) were cultured in Ham's F12 (PAA, Cölbe, Germany), 2 mM glutamine (Roth, Karlsruhe, Germany), 10% fetal calf serum (Biochrom, Berlin, Germany), 50 units/mL penicillin G (Sigma-Aldrich, Steinheim, Germany), 50 µg/mL streptomycin (Sigma-Aldrich) and seeded on 96-well cell culture plates (20000 cells/well) for luciferase assays. Cells were transfected the next day using Lipofectamin 2000 (Invitrogen, Karlsruhe, Germany) with 100 ng or the indicated amount (Fig. 6A) of each split-CreERT2 plasmid/well. For reporter-only controls, the same amount of an empty vector was transfected. For analysis of Cre-dependent recombination, a CMV-LoxP-STOP-LoxP-firefly luciferase reporter plasmid was cotransfected (100 ng/well). In addition, to normalize for transfection efficiency, a mixture of plasmids coding for renilla luciferase (total 50 ng/well; CMV-RL, TK-RL and SV40-RL at 1∶2∶10 molar ratio) was cotransfected as described [16] and the ratio of firefly luciferase activity to renilla luciferase activity was calculated. 24 h after transfection, 4OHT (Sigma-Aldrich; 1 µM or the indicated concentration (Fig. 6B)) was added and luciferase activity was measured a further 48 h later using a Lumistar microplate reader (BMG Labtech, Offenburg, Germany).

PC12 20.4 cells [16] contain a stably integrated CMV-LoxP-STOP-LoxP-EGFP cassette which enables analysis of Cre mediated DNA recombination by EGFP fluorescence. Cells were cultured in RPMI 1640 (PAA), 5% fetal calf serum, 10% horse serum, 2 mM glutamine, 50 units/mL penicillin G, 50 µg/mL streptomycin, 10 mM HEPES (Roche, Mannheim, Germany), 25 mM glucose (Roth), 15 mM NaHCO_3_ (Roth), 1 mM sodium pyruvate (Sigma-Aldrich). For experiments cells were seeded on 24-well cell culture plates (60000 cells/well) and transfected the day after plating using Lipofectamin 2000 (Invitrogen) with 400 ng or the indicated amount (Fig. 6C) of each split-CreERT2 plasmid/well. For reporter-only controls, the same amount of an empty vector was transfected. 24 h after transfection, 4OHT (1 µM or the indicated concentration (Fig. 6D)) was added. A further 48 h later, cells were harvested by trypsinization and analyzed for EGFP expression using flow cytometry (LSR II, BD Biosciences, Heidelberg, Germany).

### Western Blotting

CHO cells were seeded in 6-well culture plates (1000000 cells/well) and transfected the next day using Lipofectamin 2000 (Invitrogen) with 4 µg of each plasmid/well. For vector-transfected controls, the same amount of an empty vector was transfected. 24 h after transfection, 4OHT (1 µM) was added and cell lysates were prepared 48 h later in lysis buffer (10 mM Tris/HCl, 5 mM EDTA, pH 7.4) supplemented with Complete protease inhibitor cocktail (Roche, Mannheim, Germany). Samples were homogenized using a sonicator and protein content was assayed with BCA (Thermo Scientific, Waltham, USA). Lysates were mixed with sample buffer (375 mM Tris/HCl, pH 6.8, 5% SDS, 12% glycerol, 0.05% bromophenol blue, 10% β-mercaptoethanol) and heated for 30 min at 50°C (detection of Myc-tag) or for 10 min at 85°C (all other antibodies). 15 µg of protein per lane were separated by SDS-PAGE and blotted onto a nitrocellulose membrane (Amersham, Brussel, Belgium). Membranes were blocked with 5% milk powder and 10% Roti-Block (Roth) in PBS (pH 7.4) and incubated with the following primary antibodies: mouse anti-GAPDH (1∶1000; Ambion, Foster City, USA), mouse anti-Myc tag (1∶500; Sigma-Aldrich), HRP-conjugated mouse anti-Flag tag (1∶1000; Sigma-Aldrich), rabbit anti-Cre (1∶1000; Covance, Princeton, USA). The following secondary antibodies were used: HRP-conjugated goat anti-mouse IgG (1∶1000; Dianova, Hamburg, Germany) and HRP-conjugated goat anti-rabbit IgG (1∶1000; Santa Cruz Biotechnology, Heidelberg, Germany). All antibodies were diluted in 5% milk powder in PBS (pH 7.4).

### Immunocytochemical Analysis

For immunocytochemical analysis, CHO cells (60000 to 100000 cells/well) were cultured on glass coverslips in 24-well cell culture plates. Cells were transfected one day after plating with the following plasmids: NE and EC (150 or 400 ng each/well), a reporter plasmid expressing EGFP after Cre-mediated DNA recombination (400 ng/well) and in some cases (Fig. 5A, B) with pCMV-DsRedNuc (100 ng/well) using Lipofectamin 2000 (Invitrogen). 24 h after transfection, 4OHT (1 µM) was added. 48 h later, cells were fixed for 10 min in 4% paraformaldehyde (Fig. 5A, B) or for 10 min with 1% paraformaldehyde and 10 min with MeOH at −20°C (Fig. 5C–F). Immunostaining was performed using the following antibodies: Fig. 5A, B: rabbit anti-GFP (1∶500; Abcam, Cambridge, UK) and Cy2-conjugated goat anti-rabbit IgG (1∶1000; Dianova). Fig. 5C–F: mouse anti-Myc (1∶5000; Sigma-Aldrich); rabbit anti-Flag (1∶1000; Rockland, Gilbertsville, PA, USA), goat anti-GFP (1∶2000; Rockland); Cy3-conjugated donkey anti-mouse IgG (1∶5000; Dianova); Cy5-conjugated donkey anti-rabbit IgG (1∶400; Dianova) as well as Cy2-conjugated donkey anti-goat IgG (1∶1000; Dianova). Fluorescence images were acquired on an Axiovert 200 fluorescence microscope (Zeiss, Oberkochen, Germany) using either a Plan-Apochromat 63x/1.40 or a Fluar 40x/1.30 objective (both from Zeiss).

### Presentation of Data

Figures were generated using Sigmaplot 10.0 (Systat, Erkrath, Germany) and statistical tests were performed using SigmaStat 3.5 (Systat). All data are given as mean±standard error of the mean (SEM). Microscopic images were processed using Axiovision software (Zeiss) and Adobe Photoshop. Final illustrations were arranged using Adobe InDesign.
